# Tickle contagion in the rat somatosensory cortex

**DOI:** 10.1016/j.isci.2022.105718

**Published:** 2022-12-05

**Authors:** Lena V. Kaufmann, Michael Brecht, Shimpei Ishiyama

**Affiliations:** 1 Bernstein Center for Computational Neuroscience Berlin, Institut für Biologie, Humboldt-Universität zu Berlin, Philippstraße 13, Haus 6, 10115 Berlin, Germany; 2 NeuroCure Cluster of Excellence, Humboldt-Universität zu Berlin, Charitéplatz 1, 10117 Berlin, Germany; 3 Institut für Pathophysiologie, Universitätsmedizin der Johannes Gutenberg-Universität Mainz, Duesbergweg 6, 55128 Mainz, Germany; 4 Berlin School of Mind and Brain, Humboldt-Universität zu Berlin, Berlin, Germany

**Keywords:** Sensory neuroscience

## Abstract

The cellular mechanisms of emotional contagion are unknown. We investigated tickle contagion and the underlying neuronal representations in playful rats. We recorded trunk somatosensory cortex activity of observer rats while they received tickling and audiovisual playback of tickling footage and while they witnessed tickling of demonstrator rats. Observers vocalized and showed “Freudensprünge” (“joy jumps”) during witnessing live tickling, while they showed little behavioral responses to playbacks. Deep layers in the trunk somatosensory neurons showed a larger correlation between direct and witnessed tickling responses compared to superficial layers. Trunk somatosensory neurons discharged upon emission of own and demonstrator’s vocalizations and might drive contagious “laughter”. We conclude that trunk somatosensory cortex might represent ticklishness contagion.

## Highlights

• Observer rats vocalize and show Freudensprünge during witnessing tickling
• Air tickling and witnessing tickling activate trunk somatosensory cortex
• Demonstrator’s vocalizations activate trunk somatosensory cortex
• Direct and witnessed tickle responses are correlated in the deep layer trunk cortex

## Graphical abstract

**Figure:**
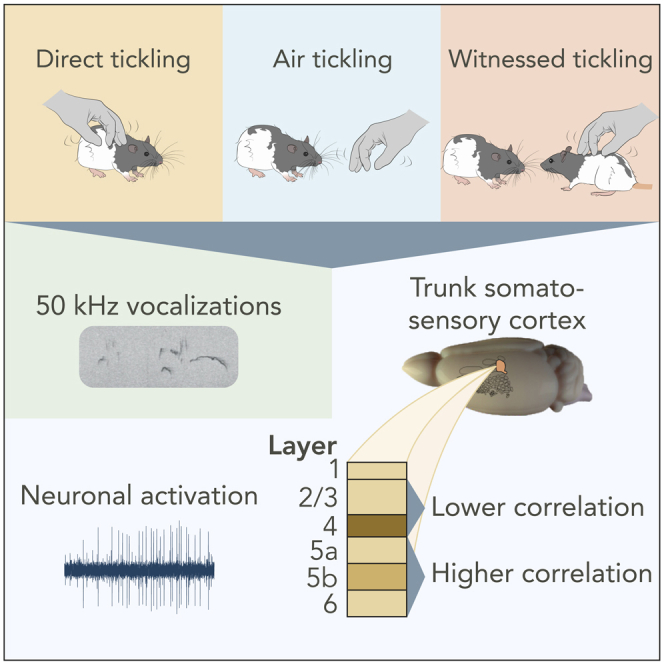

## Introduction

The capacity of an individual to share the feelings of other individuals^1^ plays a critical role in human social interactions.^2^ Possible mechanisms for this phenomenon have for a long time been a subject of discussions in various disciplines.^3^ From an evolutionary point of view, emotional contagion, “feeling into” one’s conspecifics, can be a vital ability, not only for humans but for all social species.^4^^,^^5^^,^^6^ Emotional contagion as a way to gain rapid emotional connectedness has been proposed to have its origins in parental behavior and is thought to be the root of empathy and a precursor of prosocial behavior.^7^ There is a growing field of research on rodent empathy, but until now these studies have mainly been focusing on negative emotions.^8^ With empathy being a rather ambiguous term, we want to make it clear that we only focus on its basal form of emotional contagion as “primal empathy”^1^ or “affective empathy”,^5^ rather than higher, cognitive embodiments of empathy.

In addition to the higher order visual system, mirror neurons have been suggested to be involved in understanding others’ actions.^9^ Since their first discovery in macaque ventral premotor regions as neurons responding not only to the execution but also to the observation of specific motor actions,^10^^,^^11^ mirror neurons have been described in various brain regions in macaques^12^^,^^13^^,^^14^^,^^15^^,^^16^^,^^17^ and humans.^18^ Recent studies found an involvement of mouse anterior cingulate cortex-to-nucleus accumbens projections in the social transfer of pain,^19^ of the mouse suprachiasmatic nucleus in itch contagion,^20^ and of the rat anterior cingulate cortex in experienced and witnessed pain—“emotional mirror neurons”.^21^ Interestingly, somatosensory regions have also been described to play a role in the visual recognition of emotion, and the reactivation of internal somatosensory representations is necessary to simulate the emotional state of another.^22^^,^^23^^,^^24^^,^^25^

Despite human laughter contagion being suggested by earlier works,^26^ so far there are few studies investigating whether the mere observation of others being tickled induces laughter and the brain mechanisms of possible tickle contagion. With help of advances that provided evidence of ticklishness in rats^27^ and the neural correlates of ticklishness,^28^ we investigated whether the rats’ behavioral response to tickling is contagious and whether mere observation of a demonstrator rat being tickled is sufficient to induce a measurable positive emotional state in a playful observer rat. We describe behavioral observations suggestive of “laughter contagion” or more generally contagion of playful behavior in rats. We show here that the trunk region of the somatosensory cortex represents contagion of ticklishness.

## Results

### Contagion of ticklish behaviors

**Figure 1:**
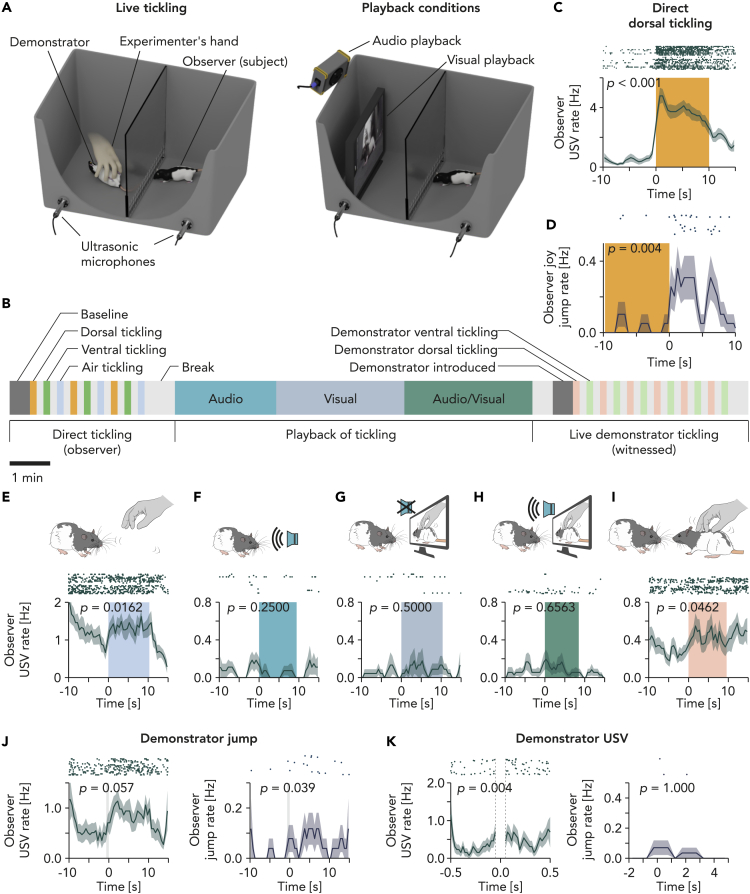
Experimental setup and behavioral response to witnessed tickling (A) Experimental setup for live demonstration (left) and audio/visual playback (right) of tickling. The box was separated by a transparent acrylic partition with a steel mesh at the bottom. (B) Timeline of a typical experimental paradigm. Color boxes indicate different event phases. Order of playback stimuli (audio; visual; audio/visual) was randomized in each recording. (C) Raster plot and peri-stimulus time histogram (PSTH) of ultrasonic vocalization (USV) rate aligned to the onset of direct dorsal tickling (0.5 s bin; mean ± SEM). Width of the color box indicates mean duration of tickling (22 events from 7 recordings). p value: signed-rank test for mean rate in [-5, −1] s vs. [1, 5] s. (D) Same as (C) but for jump (“*Freudensprung*”) rate aligned to the offset of direct dorsal tickling (13 tickling events from 4 recordings). (E) Top, schematic illustration of air tickling (hand with tickling motion without touching). Bottom, raster plot and PSTH of observer USV rate, aligned to the onset of air tickling (0.5 s bin; 21 events). p value: signed-rank test for mean rate in [-5, −1] s vs. [1, 5] s. (F) Same as (E) but for audio playback of dorsal tickling (18 events). (G) Same as (E) but for visual playback of dorsal tickling (15 events). (H) Same as (E) but for audio/visual playback of dorsal tickling (24 events). (I) Same as (E) but for witnessed dorsal tickling of demonstrator (45 events). (J) PSTHs and raster plots of observer USV (left, 17 events) and observer jump (right, 17 events) rate, aligned to the offset of demonstrator jump during break phases (no previous jump in [-10, 0] s). p value: signed-rank test for mean rate during [-9, −1] s vs. [1, 9] s. (K) Same as (J) but aligned to the onset of demonstrator USV in break phases (left, 216 events; right, 19 events). Period [-0.05, 0.05] s was excluded from the USV analyses due to analytical limitation on separation of overlapped USVs. p value: signed-rank test for mean rate during [-0.4, −0.2] s vs. [0.1, 0.3] s for USV rate and [-2, 0] s vs. [0, 5] s for jump rate, respectively.

Observer (= subject) rats were placed in a compartment of a box separated from a demonstrator compartment (Figure 1A). The observer rats received tickling on the dorsal and ventral trunk by the experimenter and air tickling (experimenter made a tickling motion in the demonstrator compartment). The observer rats then received audio, visual, and audiovisual playback of rat tickling footage (Figure 1A right). Finally, a demonstrator rat was introduced and tickled (witnessed tickling, Figure 1A, left). The experimental paradigm is illustrated in Figure 1B. The subject rats seemed to pay attention to the live demonstrator but not to the playback stimuli, quantified as fractions of time where the head was directed to the demonstrator compartment (air tickling: 80 ± 7%, n = 4, p = 0.023; audio/visual playback: 65 ± 5%, n = 4, p = 0.058; visual playback: 62 ± 10%, n = 3, p = 0.353; demonstrator introduction: 79 ± 7%, n = 4, p = 0.026; demonstrator tickled: 85 ± 8%, n = 4, p = 0.015; mean ± SEM, *n*: number of recordings from 4 rats, *p*: one-sample *t*-test with 50 as the hypothetical mean value). Differences in sound intensity of ultrasonic vocalizations (USVs) captured by four microphones, combined with positions of the animals relative to the microphones, allowed us to assign the emitter of most (90.8%) calls. As shown previously,^28^^,^^29^ the observer rats strongly responded to tickling with USVs (Figure 1C) and showed *Freudensprünge* (“joy jumps”; Figure 1D). Air tickling induced vocalizations (Figure 1E) but not jumps. Audio and/or visual playback of tickling footage had little effect on the observers’ USVs (Figures 1F–1H). Interestingly, however, the observer rats vocalized when the demonstrator rats were tickled (Figure 1I; Video S1). The observers responded also to the demonstrators’ spontaneous behaviors: vocal and jump response to demonstrator jump (Figure 1J); vocal but not jump response to demonstrator USVs (Figure 1K).

Video S1. Behavioral contagion of ticklishness, related to Figure 1

### Neuronal response to air tickling and witnessed tickling

**Figure 2:**
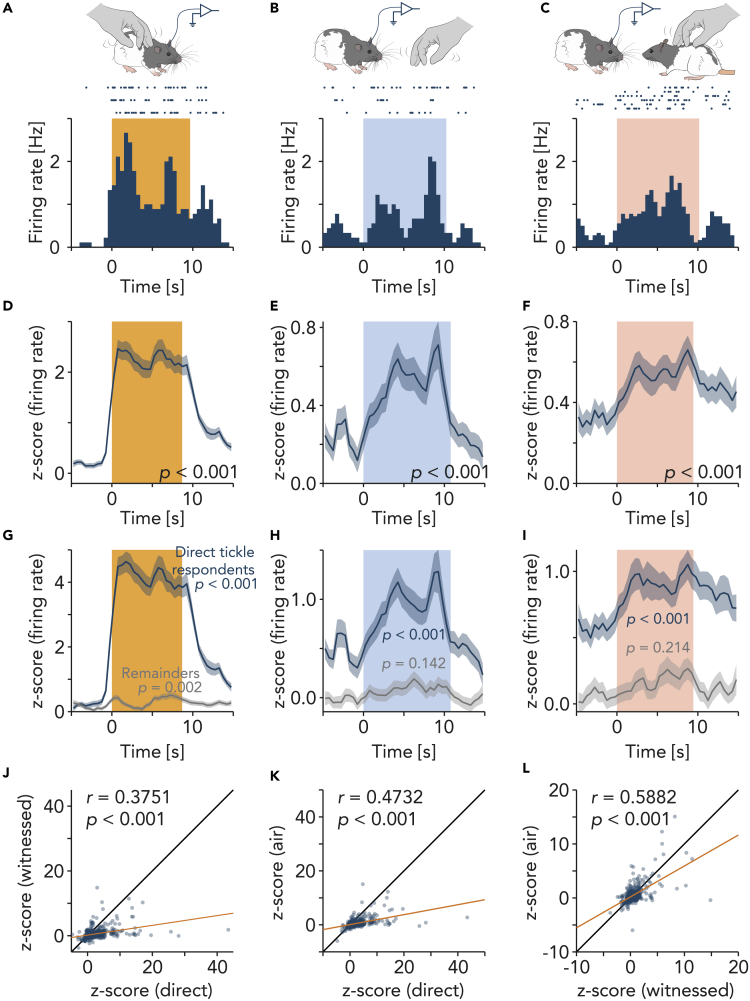
Somatosensory cortical neurons respond to tickling, air tickling, and witnessed tickling (A) Top, schematic illustration of neuronal recording during direct dorsal tickling. Bottom, raster plot and peri-stimulus time histogram (PSTH) of firing rate of a representative unit in layer 5b aligned to the onset of direct dorsal tickling (0.5 s bin). Width of the color box indicates mean duration of events. (B) Same unit response as (B) but for air tickling. (C) Same unit response as (B) but for witnessed tickling of demonstrator. (D) Population PSTH of *Z*-scored (see STAR Methods) firing rate aligned to the onset of direct dorsal tickling (Mean ± SEM; 545 units, 96 events). p value: signed-rank test for *Z* score before event onset [-5, −1] s vs. after event onset [1, 5] s. (E) Same as (D) but for air tickling (545 units, 75 events). PSTH with the same axes scaling as (D) is shown in Figure S3B. (F) Same as (D) but for witnessed dorsal tickling (545 units, 159 events). PSTH with the same axes scaling as (D) is shown in Figure S3C. (G) Same as (D) but plotted direct tickle respondent units (dark cyan, 273 units; see STAR Methods) and remainders (gray, 272 units) separately. (H) Same as (G) but for air tickling. (I) Same as (G) but for witnessed dorsal tickling. (J) Scatterplot shows mean *Z*-scored firing rate during direct dorsal tickling vs. witnessed dorsal tickling, fitted with orange line. *r*: Pearson’s correlation coefficient; *p*: p value of correlation coefficient. Black line: unity line. (K) Same as (J) but for direct dorsal tickling vs. air tickling. (L) Same as (J) but for witnessed dorsal tickling vs. air tickling.

We recorded extracellular unit activity in the observers’ trunk somatosensory cortex. An example cell in layer 5b showed a strong response to direct dorsal tickling, air tickling, and witnessed demonstrator dorsal tickling (Figures 2A–2C). We calculated a *Z* score of the firing rate based on the baseline firing rate for each unit. The firing rate at random time points in the baseline showed stable neuronal activity in the baseline (Figure S1). Population trunk somatosensory neurons responded to direct, air, and witnessed tickling, quantified by comparing *Z* scores before and after the onset of events (Figures 2D–2F and S3A–S3C). Figure S2 shows an overview of the trunk neuronal response patterns to direct, air, and witnessed tickling. Trunk somatosensory units that strongly responded to direct tickling (Figure 2G, direct tickle respondents) were strongly excited also during air tickling (Figure 2H) and witnessed tickling (Figure 2I). Population analysis showed that neuronal activities during these events were significantly correlated (Figures 2J–2L).

**Figure 3:**
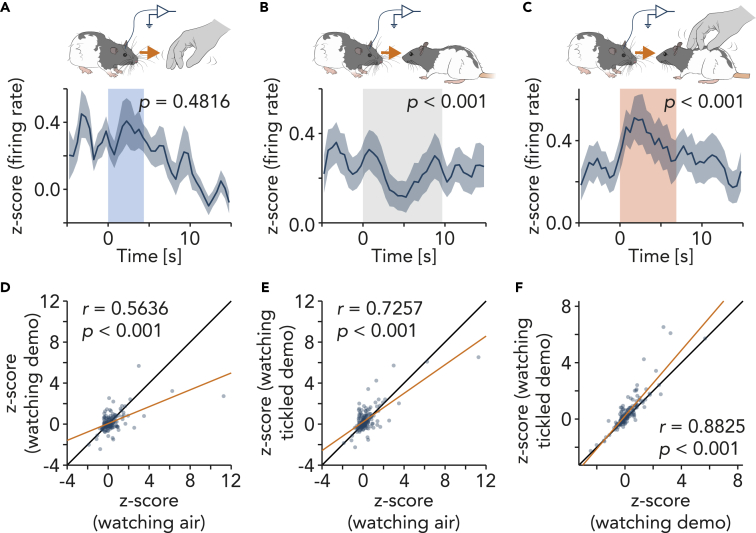
Neuronal responses to watching air tickling, watching demonstrator, and watching tickled demonstrator (A) Top, schematic illustration of neuronal recording during direct watching (turning the head toward) air tickling. Bottom, peri-stimulus time histogram (PSTH) of *Z*-scored (see STAR Methods) firing rate aligned to the onset of watching air tickling (Mean ± SEM; 148 units, 61 events). Width of the color box indicates mean duration of events. p value: signed-rank test for *Z* score before event onset [-5, −1] s vs. after event onset [1, 5] s. (B) Same as (A) but for watching untickled demonstrator (148 units, 73 events). (C) Same as (A) but for watching tickled demonstrator (148 units, 22 events). (D) Scatterplot shows mean *Z*-scored firing rate during watching air tickling vs. watching untickled demonstrator, fitted with orange line. *r*: Pearson’s correlation coefficient; *p*: p value of correlation coefficient. Black line: unity line. (E) Same as (G) but for watching air tickling vs. watching tickled demonstrator. (F) Same as (G) but for watching untickled demonstrator vs. watching tickled demonstrator.

Next, we aligned the *Z*-scored firing rate to the moment when the observers watched, i.e. turned the head toward, air tickling, the untouched demonstrator rat, and the tickled demonstrator rat. Watching air tickling did not alter the firing rate (Figure 3A), pure watching of the demonstrator inhibited the neuronal activity (Figure 3B), and watching tickling of the demonstrator increased the activity (Figure 3C). Neuronal activities during these phases were significantly correlated (Figures 3D–3F).

Trunk somatosensory neurons did not respond to playback or watching playback footage of tickling, except for watching visual playback (Figure S4).

### Stronger correlation between direct and witnessed tickling responses in deep layers

**Figure 4:**
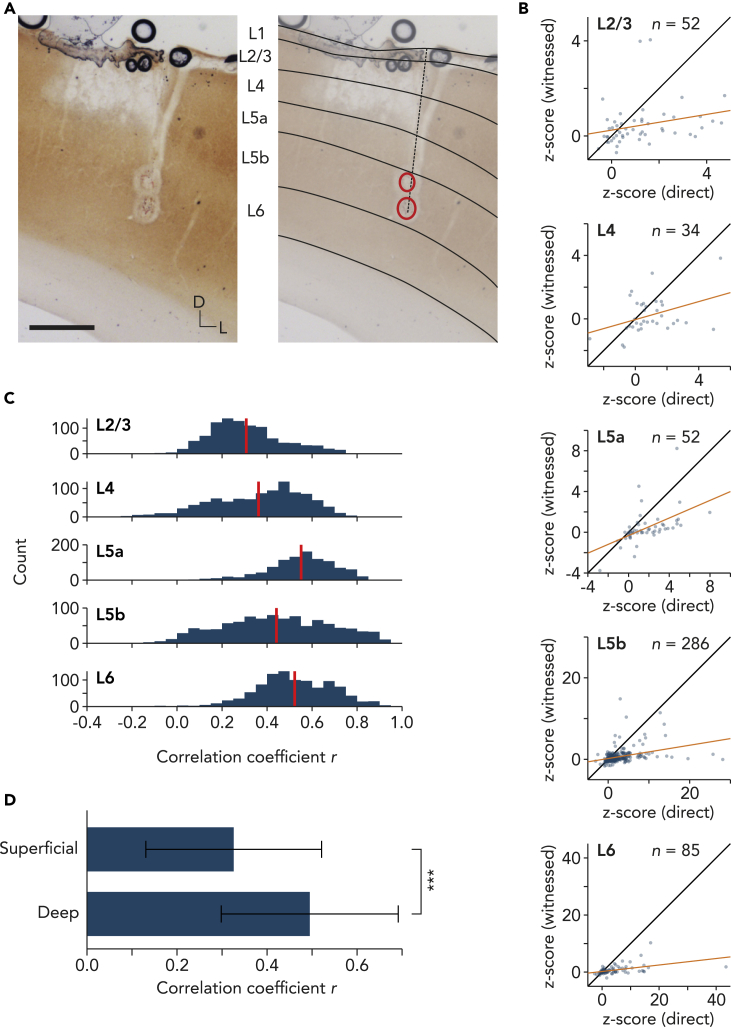
Tickle response predicts witnessed tickle response in deep-layer trunk cortex (A) Representative histological analysis for layer assignment. Left, a coronal brain section stained for cytochrome *c* oxidase (0.5 mm scale bar). Right, assignment of cortical layers (black lines). Dashed line: tetrode track. Red circles: lesions. D: dorsal. L: lateral. (B) Scatterplots show *Z*-scored firing rate during dorsal tickling (direct) vs. witnessed dorsal tickling of demonstrator (witnessed) in layers 2/3, 4, 5a, 5b, and 6. Black diagonal lines are unity lines. Orange lines are linear fit. *n*: number of units. (C) Histogram shows distribution of Pearson’s correlation coefficient *r* calculated for 1,000 times with randomly chosen 20 units, i.e. 60% of layer 4 sample size, in each layer. Red lines: mean. *p* < 0.001 (Kruskal-Wallis test). (D) Comparison of correlation coefficients in superficial (L2-4) vs. deep (L5-6) layers. Error bars: SD. *p* < 0.001 (rank-sum test).

We next analyzed the neuronal responses across cortical layers. Cortical layers were assigned according to cytochrome *c* oxidase staining (Figure 4A). *Z*-scored firing rates during direct and witnessed tickling were calculated for each layer (Figure 4B). Because sample numbers were largely different across layers, we randomly selected 20 units in each layer for the calculation of the correlation coefficient and repeated this 1,000 times (Figure 4C), revealing a stronger correlation in deep layers (5a; 5b; 6) compared to the superficial layers (2/3; 4) (Figure 4D). This was also the case for direct and air tickling responses, but here we also found a strong correlation in layer 4 (Figure S5). The difference between superficial and deep layers in the correlation of witnessed tickling and air tickling was more prominent (Figure S6).

### Trunk cortex responds to own and demonstrator’s vocalizations

**Figure 5:**
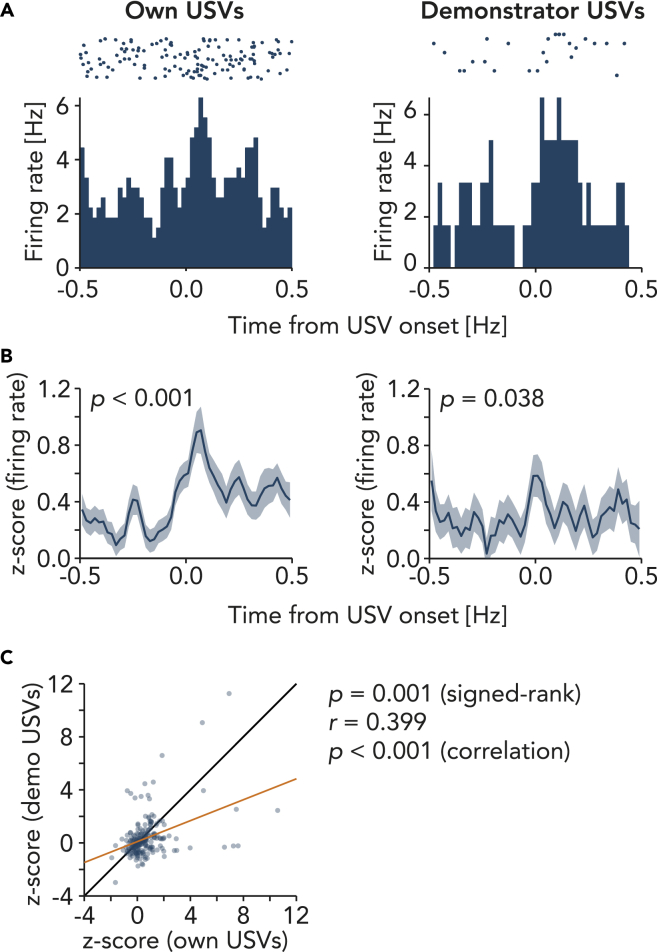
Trunk cortical neurons discharge during own and demonstrator vocalizations (A) Representative raster and peri-stimulus time histograms (PSTHs) of a unit in layer 5b trunk somatosensory cortex, aligned to the onset of own (observer’s) ultrasonic vocalizations (USVs, in break phases with no previous USV onset within [-0.5, 0] s, left) and demonstrator USVs (right). Data are binned to 20 ms. (B) Population PSTHs of *Z*-scored firing rate of 210 units (mean ± SEM) aligned to own USV onset (left, 566 USVs) and demonstrator USV onset (right, 216 USVs). p value: signed-rank test for *Z* score before USV onset [-0.4, −0.2] s vs. after USV onset [0.0, 0.2] s. (C) Scatterplot shows mean *Z*-scored firing rate during own USVs (B, left) vs. demonstrator USVs (B, right). Black line: unity line. Orange line: linear fit. *r*: Pearson’s correlation coefficient.

We previously showed that the trunk somatosensory neurons increase their firing rate prior to and during USV emissions, even in the absence of tickling.^28^ We compared the observers’ neuronal response to own and demonstrators’ USV emission during the breaks (i.e. out of tickling; Figure 5A, representative unit in L5b). Population analysis showed that the trunk somatosensory cortex neurons responded weakly yet significantly also to demonstrator USVs (Figure 5B). Neuronal responses to own and demonstrator USVs were significantly correlated (Figure 5C).

## Discussion

### Playfulness contagion

We verified our previous results^28^ that rats show a neuronal and behavioral response to tickling. Playful rats showed interest in the conspecifics being tickled. Fifty-kHz USVs are reliable measures of positive emotions in rats^30^ and *Freudensprünge* are described as a behavior that is seen in joyful rats^28^^,^^31^ and other species including piglets,^32^ dogs,^33^ foxes,^34^ and guinea pigs.^35^ We found such playful behaviors in the observer rat witnessing the demonstrator rat being tickled, demonstrator jump, and demonstrator USVs, pointing to an emotional contagion of playfulness. This kind of playfulness contagion has been observed also in other species, such as ravens^36^ and keas.^37^ Contagion of laughter has been suggested in humans^38^ and chimpanzees.^39^ Several studies on rats showed an increase in play by introduction of a more playful individual^40^^,^^41^ and tickling-induced emotional contagion from a tickled rat to their cage mate.^42^ Devocalized pairs of rats show reduced play frequency.^43^ Artificially deafened but not blinded rats^44^ play less than their conspecifics.^45^

We found that the observer rat showed little behavioral response to audio and/or visual playback of tickling footage (Figures 1F–1H) but more to the live demonstrator rat being tickled suggesting vicarious playfulness. It is remarkable that playfulness contagion is not merely carried by the sound and vision, but it requires the presence of a live demonstrator. In fact, we occasionally noticed that the observers were sleeping during playback. Yet, it remains to be tested how differently playback and live demonstration of play lead to an increase in play behavior in observer animals. Our experimental setup (Figure 1A) had a partition with metal mesh at the bottom so that the rats could smell each other. The striking difference in the behavioral responses to witnessing live vs. audio/visual playback tickling may suggest an important role of olfactory components in playfulness contagion.

Rats responded to air tickling (a hand with tickling motion) with USVs, suggesting an anticipation of tickling. One could argue whether the response to witnessed tickling is merely an anticipatory response to the hand, rather than playfulness contagion. We consider that the observers’ response to witnessed tickling is not a response just to the hand because the observers responded also to demonstrators’ spontaneous behaviors such as jump and USVs when the experimenter’s hand was not in the demonstrator compartment (Figures 1J and 1K). Because air tickling was administered shortly after direct tickling (Figure 1B), the rats were already vocalizing prior to air tickling, which would overestimate the effect of air tickling (Figure 1E). In contrast, the demonstrator was tickled a long time after direct tickling, and even after playback of tickling, which would have led to an underestimation of the response to witnessed tickling when compared with air tickling.

### Cortical response to witnessed tickling and anticipatory response

Next, we found that trunk somatosensory neurons responded to both direct and witnessed tickling (Figure 2), suggesting that somatosensory cortex represents playfulness contagion, or possibly “tickling mirror-like” neurons. Moreover, this neuronal response was associated with positive emotional behavior (Figure 1I). Many units that responded to direct tickling were also excited by air tickling (Figures 2H, 2J, and S4), however. Responses to air tickling and witnessed tickling were considerably smaller than response to direct tickling (Figure S3). Accordingly, it seems also possible that these units responded rather to the expectancy of being tickled than the observed tickling act itself, and we think this possibility cannot be fully excluded. In fact, individual neurons that were strongly activated during witnessing tickling certainly gave the impression of true “mirroring”. The most likely expectancy-driven responses seen in control conditions (air tickling) leave us unable to decide whether at the population level our responses represent true mirroring or play expectancy. Previous studies also found that somatosensory neurons fire in response to expected tickling during hand chase^28^ and slow hand approach.^29^ We note that few mirroring studies used the type of strong control conditions that we applied (air tickling) and that further work may help to differentiate mirroring and (play-)expectancy. Yet, there is precedent to the idea that the mirror neuron system implements anticipatory and simulative capabilities, i.e. an internal simulation and anticipation of the consequences of an action.^46^^,^^47^^,^^48^^,^^49^ Anderson & Adolphs^50^ emphasized that persistence is a characteristic feature of emotional processing in comparison with other sensory processings. Activity of trunk somatosensory neurons, particularly the direct-tickle respondents (Figure 2), increased during direct tickling, decreased but remained elevated from the baseline during break, and increased by witnessing tickling. This persistence and revoking in the activity without a tactile stimulus could be better explained as emotional processing, rather than tactile processing. Previous experience of being tickled might also play a role in tickle contagion. Thus, experiments using tickle-naïve observer rats would be of interest to test whether ticklishness is innate or acquired. We conclude that somatosensory cortical neurons show strong visual responses that are driven by a mirroring or play-expectancy mechanism and that contribute to tickle contagion.

We found that the correlation between the direct and the witnessed tickling response was stronger in deep layers compared to superficial layers (Figure 4). Traditionally, the somatosensory cortex is seen as a sensory map with layer 4 being a sensory relay center, but recently we proposed a broader capability of the somatosensory system as a body model that is not only receiving and relaying sensory inputs but serves as a representation of the body and body simulation.^51^ We previously showed that discharge of layer 4 trunk somatosensory neurons is sharply aligned to the onset of tickling, whereas layer 5 plays a role in anticipation of tickling i.e. activation prior to tickling onset.^29^ Layer 5 neurons also show strong responses during tickling, and microstimulation of deep layers evokes USVs.^28^ The role of deep layers of the trunk somatosensory cortex in tickling and witnessing tickling could indicate that the deep layers are more related to emotional states. Interestingly, laminar pattern of correlation of direct tickling and air tickling response (Figure S5) as well as correlation of witnessed tickling and air tickling (Figure S6) was different from that of direct and witnessed tickling, which might suggest different mechanisms underlying direct, air, and witnessed tickling responses.

In line with a previous report,^28^ trunk somatosensory neurons discharged upon vocal emission (Figure 5). The neurons weakly yet significantly responded also to USVs emitted by the demonstrator rats during break phases i.e. out of tickling. Playback of 50-kHz USVs is reported to induce response calls,^52^ social approach,^53^ and increased c-fos expression in the frontal cortex and the nucleus accumbens.^54^ Whereas our audio playback experiment did not induce observer’s response, USVs emitted by the live demonstrator out of tickling led to USV emission (Figure 1K) and activation of trunk somatosensory cortex (Figure 5) in the observer animals. These results further support that the behavioral and neuronal response to vicarious tickling is a response not only to the hand but also to the exhilarated emotional state of the demonstrator rats. Yet, our conclusion is limited to the observation of the somatosensory cortex. We do not know to what extent the trunk somatosensory cortex has a role in emotional contagion, given that many other brain regions play a more prominent role in emotions than the somatosensory cortex. Thus, further work including investigation of other brain areas is needed to understand this interesting behavior.

Trunk somatosensory neurons showed little if not no response to the audio and/or visual playback of conspecific tickling (Figure S4). There are actually many accounts of modality-specific mirror neurons. At least two studies found auditory mirror neurons in macaques,^55^^,^^56^ and neuroimaging in humans led to the proposition of a somatotopic auditory mirror system. Most of this proposed system, however, appears to be multimodal.^57^ Recently, a study revealed modulation of the barrel cortex by the superior colliculus in mice.^58^ Regarding the rat ecology as nocturnal and rats as not very visual animals that are prey for a number of hunters and rely more on their hearing and olfaction,^59^ more cells might respond to auditory stimuli, or these responses could be stronger than responses to other sensory inputs. Anatomical tracing experiments could potentially reveal more about multisensory integration in the somatosensory cortex.

A recent study demonstrated that rats distinguish a USV emitter, and they self-administer preferentially playback of 50-kHz USVs emitted by a stranger rat over those emitted by their cage mate.^60^ We used playback footage of a rat that was a stranger to the observers, whereas the live demonstrators were familiar to the observers. Using live demonstration of a stranger rat, therefore, might have been a more rewarding stimulus to the observer rats. It is to be noted, however, that familiarity with the demonstrator is crucial in empathetic behavior of the observer in rodents, at least for negative experiences.^61^^,^^62^^,^^63^^,^^64^

Carrillo et al. showed that there are emotional mirror neurons in the rat anterior cingulate cortex.^21^ The central amygdala also appears to be involved in the recognition and emotional contagion of socially signaled danger.^65^^,^^66^ It is likely that the response to direct and witnessed tickling that we observed in the somatosensory cortex neurons was strongly influenced by or dependent on the positive emotional valence of the tickling. Interestingly, a number of studies report that the recognition of emotional expression in faces^22^^,^^67^ and voices^68^^,^^69^ depends on the right somatosensory cortex in humans. Furthermore, it has been proposed that the somatosensory cortex plays a role in linking the perception of emotional expressions with subjective sensory experience.^23^

Taken together, our results suggest behavioral and neuronal contagion of ticklish “laughter” and playfulness in playful rats. Hence, our research opens new avenues to investigate positive emotions, which are rather understudied in neuroscience. We acknowledge that more work needs to be done on contagious laughter in humans and rats. Much like in humans, live performance (i.e. demonstrator tickling) was much more engaging in rats than the various forms of playback (i.e. watching TV). Mapping of others’ body representations on a “body model” in the somatosensory cortex,^51^ facilitated by emotional valence, would be a possible explanation for these findings.

### Limitation of the study

Our observations apply to tickle-experienced playful individuals. Demonstrators were familiar with observers. Air tickling was delivered shortly after direct tickling, whereas witnessed tickling was administrated long after direct tickling.

## STAR★Methods

### Key resources table

**Table:** 

REAGENT or RESOURCE	SOURCE	IDENTIFIER
**Experimental models: Organisms/strains**		

Rat: RjOrl:LE	Janvier Labs	https://janvier-labs.com/en/fiche_produit/long-evans_rat/

**Software and algorithms**		

MATLAB	Mathworks	https://www.mathworks.com/products/matlab.html
Python 2.7 & 3.6	Python	https://www.python.org/
Cheetah	Neuralynx	https://neuralynx.com/software/cheetah
Avisoft RECORDER USGH	Avisoft Bioacoustics	https://www.avisoft.com/recorder.htm
MClust 3.5	David Redish	https://redishlab.umn.edu/mclust
ELAN 5.1	(Lausberg and Sloetjes, 2009)	https://archive.mpi.nl/tla/elan

### Resource availability

#### Lead contact

Further information and requests for resources and reagents should be directed to and will be fulfilled by the lead contact, Shimpei Ishiyama (shimpei.ishiyama@uni-mainz.de).

#### Materials availability

This study did not generate new unique reagents.

### Experimental model and subject details

#### Animals

Nine 3-week-old male Long-Evans rats were acquired from Janvier Labs. Animals were individually housed, maintained with a 12:12 h inverted light/dark circle and allowed *ad libitum* access to food and water. Prior to the experiments, animals were handled for 20 minutes and tickled for 10 minutes^28^ individually once a day for 6–8 days. Additionally, the rats were assigned to 3 pairs and 1 trio for later observer/demonstrator roles. In one case, one demonstrator rat was used as a partner for two observer rats in their respective experiments. Thus, we used 5 subjects (observers) and 4 demonstrators. The rats were accustomed to the experimental setup and allowed to interact with their partner conspecifics for 20 minutes every day during the habituation period to avoid any novelty effect later during the experiment. At the end of the habituation period, rats responding with more jumps and more ultrasonic vocalizations (USVs) to tickling were chosen as observer rats to maximize direct ticklishness observation, and less responsive ones as the demonstrator rats. The observer was not selected based on response to witnessed tickling. Experiments on one out of 5 subject rats and experiments on the other 4 subject rats were conducted by different experimenters. All habituations and experiments were conducted during the dark phase in a dark room. All experimental procedures were performed according to German guidelines on animal welfare under the supervision of local ethics committees in accordance with the animal experimentation permit (Permit No. G0193/14 and Permit No. G0279/18).

### Method details

#### Experimental setup

The experimental setup consisted of a box (‘tickle box’) lined with black foam rubber, with a plexiglass separation wall in the middle. The bottom of the separation wall was a mesh wire stripe, allowing the animals to see, hear and smell each other. The experimental environment was kept dim (∼20 lx). Video material was recorded under infrared illumination with two or three cameras: a top view and a side view camera with 30 fps (Imaging Source, Germany), and a side view with 240 fps (H5PRO – modified GoPro Hero 5, Back-bone, Ottawa, Canada). Ultrasonic vocalizations were recorded with four microphones (condenser ultrasound CM16/CMPA, frequency range 10–200 kHz, Avisoft Bioacoustics, Berlin, Germany) at a sampling rate of 250 kHz and with a 16-bit resolution using Avisoft-RECORDER USGH software (Avisoft Bioacoustics, Berlin, Germany). The microphone positioning enabled assigning calls to demonstrator and observer rat, respectively. The audio recording, video recording and electrophysiological recording were synchronized using TTL pulses. The setup is illustrated in Figure 1A.

#### Experimental paradigm

Rats were tickled as previously described.^28^ The experiments started with the observer rat alone, positioned in the left compartment of the tickle box. A 30-s baseline phase was followed by three rounds of dorsal tickling, ventral tickling, and air tickling (tickling motion in the right compartment as a control for the presence of the hand). Each phase of tickling lasted for 10 s interleaved by 15 s breaks. This was followed by playback of rat tickling footage (dorsal and ventral tickling; 3–9 repetitions), which was either only audio, only visual or audio-visual playback. For the latter two, a small display (12 inch, Beetronics, Düsseldorf, Germany) was placed in the right compartment facing the observer rat. The order of the 3 playback phases was randomized in every recording. For the last part of the experiment, the demonstrator rat was introduced and after 30 s, six rounds of dorsal and ventral tickling of the demonstrator rat i.e. witnessed tickling were performed. The procedure is illustrated in Figure 1B.

#### Surgery

Harlan 8 microdrives (Neuralynx, Bozeman, MT, USA) with 8 individually-movable tetrodes were implanted in the left trunk somatosensory cortex of the observer rats. Tetrodes were made of nichrome wire (RO800, ¼ Hard Pac, 0.0005″, Sandvik, Sweden) and gold plated to 250 kOhm impedance. Tetrodes were arranged in a 2-by-4 matrix with 0.5 mm distance in the microdrive. Animals were initially anesthetized with isoflurane (cp-pharma, Burgdorf, Germany) followed by i.p. injection of ketamine/xylazine hydrochloride solution (ketamine 80 mg/mL, xylazine 6 mg/mL solution; 1.1 μL/g body weight dosage; Sigma-Aldrich). Lidocaine was subcutaneously injected in the scalp and carprofen (10x diluted in saline; 1 μL/g dosage; Zoetis, Germany) was injected s.c. prior to incision. Booster ketamine doses (100 mg/mL solution; 0.5 μL/g body weight dosage) were administered as required. After scraping the skull, 2–3 stainless screws and a gold-plated screw with a silver wire soldered were fixed in the skull as anchors and ground, respectively. The skull was then treated with Optibond (Kerr Italia, Salerno, Italy), and Charisma dental filling (Heraeus Kulzer, Hanau, Germany). Following craniotomy (1 mm posterior, 2–4 mm lateral from Bregma) and durotomy, the microdrive was positioned on the brain and the exposed brain area was covered with 0.4% agarose. The silver wire of the gold screw was connected to the ground of the microdrive. The microdrive was secured with dental cement (Paladur, Heraeus Kulzer, Hanau, Germany). At the end of the surgery, carprofen (the same dosage as above) was injected s.c. before the animal woke up.

#### Electrophysiological recording

Starting two to three days after surgery, tetrodes were lowered into the brain by ∼0.25 mm daily. When spikes were observed, experiments started at least 1 h after lowering the tetrodes to stabilize tissue drift. Extracellular spikes in the left trunk somatosensory cortex of the observer animal were recorded at 32 kHz sampling rate and bandpass-filtered between 0.6 and 6 kHz using Cheetah software, and DigitalLynx SX (Neuralynx, Bozeman, MT, USA).

#### Histology

After the last experiment, animals were anaesthetized and the tetrode tracks were labeled with electrolytic lesions by applying a DC current (8 s, 8 μA, electrode tip negative; using nanoZ, Neuralynx, Bozeman, MT, USA; Figure 4A). Animals received an overdose of isoflurane and were transcardially perfused with a pre-fixative solution followed by a 4% paraformaldehyde solution. For histological processing, brains were cut in 80–100 μm coronal sections and stained for cytochrome-oxidase activity.^70^ Assignment of recording sites to layers was done based on the layer-specific staining.

### Quantification and statistical analysis

#### Video analysis

Video material was analyzed using ELAN 5.1 software^71^ to label durations of the experimental paradigm phases as well as rat behaviors. An ethogram used for the video analysis is shown in Table S1.

#### Ultrasonic vocalization analysis

The recorded ultrasonic vocalizations (USVs) were analyzed using a custom-written software^72^ and visually corrected by experimenters. Analysis of the USV emitter was performed by comparing sound levels of each USV in the four microphones and combining this with the top view video of the experiment to visually identify the emitter, based on the rats’ head direction and proximity to the microphones. Emitter of each USV was labelled as either ‘observer, ‘playback’, ‘demonstrator, or ‘unclear’ (particularly in case the USVs were emitted while snouts of both rats were touching). All USVs we observed were 50-kHz appetitive calls, and no 22-kHz aversive calls were detected.

#### Electrophysiological analyses

Spike sorting and clustering were performed using MClust (AD Redish, MN, USA) as described previously.^72^ Energy, time, and first two derivatives of energy of spikes were used for sorting. Units with average firing rate <0.1 Hz were excluded. Units that lack temporal stability across the recording were excluded. For population analyses, firing rates were normalized in terms of z-score, based on mean and standard deviation of firing rate (0.5 s bin) in the baseline period (first 30 s of the recording), i.e. z-score = (FR – FR_BL_) / σ_BL_, where FR is firing rate at a given bin, FR_BL_ is a mean firing rate of the same unit during the baseline, σ_BL_ is a standard deviation of the baseline firing rate. Accordingly, units with no spike during the baseline were excluded from the analysis. All peristimulus time histograms (PSTHs) were smoothed with moving average over 3 bins. To quantify population neuronal response to an event, mean z-score before and after aligned time were calculated in each unit, and comparison was tested using signed-rank test. To plot PSTHs for direct tickle-respondents vs. remainders (Figures 2G–2I), relative change in z-score was calculated i.e. mean z-score before direct dorsal tickling ([-5, −1] s) was subtracted from mean z-score during direct dorsal tickling ([1, 5] s) for each unit (Figure 2D). Top half of units with the largest change in z-score were defined as direct tickle respondents (273 units), and the other half was defined as remainders (272 units). Correlation between responses to two different events were performed by calculating Pearson’s correlation coefficient of mean ‘post’ z-scores of the events. To compare correlation coefficients between cortical layers, since number of units recorded in different layers were different (Figure 4B), 20 units were randomly selected, and correlation coefficient was calculated. This was repeated for 1,000 times and correlation coefficients were compared across layers using Kruskal-Wallis test (Figures 4C, S5B, and S6B).

#### Statistical analysis in general

Intergroup comparisons were performed with signed-rank test for paired data, and rank-sum test for unpaired data. *n* refers to sample size. Data were analyzed using MATLAB 2017b and 2018b, and Python 2.7 and 3.6.

## Data Availability

• Data reported in this paper will be shared by the lead contact upon request. • This paper does not report original code. • Any additional information required to reanalyze the data reported in this paper is available from the lead contact upon request.

## References

[1] Panksepp J., Panksepp J.B. (2013). Toward a cross-species understanding of empathy. Trends Neurosci..

[2] Hatfield E., Cacioppo J.T., Rapson R.L. (1993). Emotional contagion. Curr. Dir. Psychol. Sci..

[3] Darwin C. (1871).

[4] Nakahashi W., Ohtsuki H. (2015). When is emotional contagion adaptive?. J. Theor. Biol..

[5] de Waal F.B.M., Preston S.D. (2017). Mammalian empathy: behavioural manifestations and neural basis. Nat. Rev. Neurosci..

[6] Paradiso E., Gazzola V., Keysers C. (2021). Neural mechanisms necessary for empathy-related phenomena across species. Curr. Opin. Neurobiol..

[7] de Waal F.B. (2008). Putting the altruism back into altruism: the evolution of empathy. Annu. Rev. Psychol..

[8] Meyza K.Z., Bartal I.B.A., Monfils M.H., Panksepp J.B., Knapska E. (2017). The roots of empathy: through the lens of rodent models. Neurosci. Biobehav. Rev..

[9] Rizzolatti G., Fogassi L., Gallese V. (2001). Neurophysiological mechanisms underlying the understanding and imitation of action. Nat. Rev. Neurosci..

[10] Di Pellegrino G., Fadiga L., Fogassi L., Gallese V., Rizzolatti G. (1992). Understanding motor events: a neurophysiological study. Exp. Brain Res..

[11] Gallese V., Fadiga L., Fogassi L., Rizzolatti G. (1996). Action recognition in the premotor cortex. Brain.

[12] Fogassi L., Ferrari P.F., Gesierich B., Rozzi S., Chersi F., Rizzolatti G. (2005). Parietal lobe: from action organization to intention understanding. Science.

[13] Shepherd S.V., Klein J.T., Deaner R.O., Platt M.L. (2009). Mirroring of attention by neurons in macaque parietal cortex. Proc. Natl. Acad. Sci. USA.

[14] Tkach D., Reimer J., Hatsopoulos N.G. (2007). Congruent activity during action and action observation in motor cortex. J. Neurosci..

[15] Ishida H., Nakajima K., Inase M., Murata A. (2010). Shared mapping of own and others' bodies in visuotactile bimodal area of monkey parietal cortex. J. Cogn. Neurosci..

[16] Fujii N., Hihara S., Iriki A. (2007). Dynamic social adaptation of motion-related neurons in primate parietal cortex. PLoS One.

[17] Breveglieri R., Vaccari F.E., Bosco A., Gamberini M., Fattori P., Galletti C. (2019). Neurons modulated by action execution and observation in the macaque medial parietal cortex. Curr. Biol..

[18] Farina E., Borgnis F., Pozzo T. (2020). Mirror neurons and their relationship with neurodegenerative disorders. J. Neurosci. Res..

[19] Smith M.L., Asada N., Malenka R.C. (2021). Anterior cingulate inputs to nucleus accumbens control the social transfer of pain and analgesia. Science.

[20] Yu Y.Q., Barry D.M., Hao Y., Liu X.T., Chen Z.F. (2017). Molecular and neural basis of contagious itch behavior in mice. Science.

[21] Carrillo M., Han Y., Migliorati F., Liu M., Gazzola V., Keysers C. (2019). Emotional mirror neurons in the rat's anterior cingulate cortex. Curr. Biol..

[22] Adolphs R., Damasio H., Tranel D., Cooper G., Damasio A.R. (2000). A role for somatosensory cortices in the visual recognition of emotion as revealed by three-dimensional lesion mapping. J. Neurosci..

[23] Kragel P.A., LaBar K.S. (2016). Somatosensory representations link the perception of emotional expressions and sensory experience. eNeuro.

[24] Keysers C., Kaas J.H., Gazzola V. (2010). Somatosensation in social perception. Nat. Rev. Neurosci..

[25] Damasio A.R. (1996). The somatic marker hypothesis and the possible functions of the prefrontal cortex. Philos. Trans. R. Soc. Lond. B Biol. Sci..

[26] Provine R.R. (2004). Laughing, tickling, and the evolution of speech and self. Curr. Dir. Psychol. Sci..

[27] Panksepp J., Burgdorf J., Hameroff S.R., Chalmers D., Kaszniak A.W. (1999). Toward a Science of Consciousness III.

[28] Ishiyama S., Brecht M. (2016). Neural correlates of ticklishness in the rat somatosensory cortex. Science.

[29] Ishiyama S., Kaufmann L.V., Brecht M. (2019). Behavioral and cortical correlates of self-suppression, anticipation, and ambivalence in rat tickling. Curr. Biol..

[30] Knutson B., Burgdorf J., Panksepp J. (2002). Ultrasonic vocalizations as indices of affective states in rats. Psychol. Bull..

[31] Reinhold A.S., Sanguinetti-Scheck J.I., Hartmann K., Brecht M. (2019). Behavioral and neural correlates of hide-and-seek in rats. Science.

[32] Newberry R.C., Wood-Gush D.G., Hall J.W. (1988). Playful behavior of piglets. Behav. Processes.

[33] Käufer M. (2014).

[34] Bekoff M. (1974). Social play and play-soliciting by infant canids. Am. Zool..

[35] Harrup A.J., Rooney N. (2020). Current welfare state of pet Guinea pigs in the UK. Vet. Rec..

[36] Osvath M., Sima M. (2014). Sub-adult ravens synchronize their play: a case of emotional contagion. Anim. Behav. Cogn..

[37] Schwing R., Nelson X.J., Wein A., Parsons S. (2017). Positive emotional contagion in a New Zealand parrot. Curr. Biol..

[38] Provine R.R. (1992). Contagious laughter: laughter is a sufficient stimulus for laughs and smiles. Bull. Psychon. Soc..

[39] Davila-Ross M., Allcock B., Thomas C., Bard K.A. (2011). Aping expressions? Chimpanzees produce distinct laugh types when responding to laughter of others. Emotion.

[40] Pellis S.M., McKenna M.M. (1992). Intrinsic and extrinsic influences on play fighting in rats: effects of dominance, partner's playfulness, temperament and neonatal exposure to testosterone propionate. Behav. Brain Res..

[41] Varlinskaya E.I., Spear L.P., Spear N.E. (1999). Social behavior and social motivation in adolescent rats: role of housing conditions and partner's activity. Physiol. Behav..

[42] Hammond T., Bombail V., Nielsen B.L., Meddle S.L., Lawrence A.B., Brown S.M. (2019). Relationships between play and responses to tickling in male juvenile rats. Appl. Anim. Behav. Sci..

[43] Kisko T.M., Euston D.R., Pellis S.M. (2015). Are 50-khz calls used as play signals in the playful interactions of rats? III. The effects of devocalization on play with unfamiliar partners as juveniles and as adults. Behav. Processes.

[44] Bierley R.A., Hughes S.L., Beatty W.W. (1986). Blindness and play fighting in juvenile rats. Physiol. Behav..

[45] Siviy S.M., Panksepp J. (1987). Sensory modulation of juvenile play in rats. Dev. Psychobiol..

[46] Pezzulo G., Hoffmann J., Falcone R. (2007). Anticipation and anticipatory behavior. Cogn. Process..

[47] Maldonato M., Dell’Orco S. (2013). Mirror neurons and the predictive mind. Prog. Neurosci..

[48] Gallese V., Goldman A. (1998). Mirror neurons and the simulation theory of mind-reading. Trends Cogn. Sci..

[49] Gallese V. (2009). Mirror neurons, embodied simulation, and the neural basis of social identification. Psychoanal. Dialog..

[50] Anderson D.J., Adolphs R. (2014). A framework for studying emotions across species. Cell.

[51] Brecht M. (2017). The body model theory of somatosensory cortex. Neuron.

[52] Berz A.C., Wöhr M., Schwarting R.K.W. (2021). Response calls evoked by playback of natural 50-kHz ultrasonic vocalizations in rats. Front. Behav. Neurosci..

[53] Wöhr M., Schwarting R.K.W. (2007). Ultrasonic communication in rats: can playback of 50-kHz calls induce approach behavior?. PLoS One.

[54] Sadananda M., Wöhr M., Schwarting R.K.W. (2008). Playback of 22-kHz and 50-kHz ultrasonic vocalizations induces differential c-fos expression in rat brain. Neurosci. Lett..

[55] Kohler E., Keysers C., Umiltà M.A., Fogassi L., Gallese V., Rizzolatti G. (2002). Hearing sounds, understanding actions: action representation in mirror neurons. Science.

[56] Keysers C., Kohler E., Umiltà M.A., Nanetti L., Fogassi L., Gallese V. (2003). Audiovisual mirror neurons and action recognition. Exp. Brain Res..

[57] Gazzola V., Aziz-Zadeh L., Keysers C. (2006). Empathy and the somatotopic auditory mirror system in humans. Curr. Biol..

[58] Gharaei S., Honnuraiah S., Arabzadeh E., Stuart G.J. (2020). Superior colliculus modulates cortical coding of somatosensory information. Nat. Commun..

[59] Burn C.C. (2008). What is it like to be a rat? Rat sensory perception and its implications for experimental design and rat welfare. Appl. Anim. Behav. Sci..

[60] Vielle C., Montanari C., Pelloux Y., BAUNEZ C. (2021). Evidence for a vocal signature in the rat and its reinforcing effects. bioRxiv.

[61] Gonzalez-Liencres C., Juckel G., Tas C., Friebe A., Brüne M. (2014). Emotional contagion in mice: the role of familiarity. Behav. Brain Res..

[62] Rogers-Carter M.M., Djerdjaj A., Culp A.R., Elbaz J.A., Christianson J.P. (2018). Familiarity modulates social approach toward stressed conspecifics in female rats. PLoS One.

[63] Cox S.S., Reichel C.M. (2020). Rats display empathic behavior independent of the opportunity for social interaction. Neuropsychopharmacology.

[64] Langford D.J., Crager S.E., Shehzad Z., Smith S.B., Sotocinal S.G., Levenstadt J.S., Chanda M.L., Levitin D.J., Mogil J.S. (2006). Social modulation of pain as evidence for empathy in mice. Science.

[65] Andraka K., Kondrakiewicz K., Rojek-Sito K., Ziegart-Sadowska K., Meyza K., Nikolaev T., Hamed A., Kursa M., Wójcik M., Danielewski K. (2021). Distinct circuits in rat central amygdala for defensive behaviors evoked by socially signaled imminent versus remote danger. Curr. Biol..

[66] Keysers C., Gazzola V. (2021). Emotional contagion: improving survival by preparing for socially sensed threats. Curr. Biol..

[67] Pitcher D., Garrido L., Walsh V., Duchaine B.C. (2008). Transcranial magnetic stimulation disrupts the perception and embodiment of facial expressions. J. Neurosci..

[68] Adolphs R. (2002). Neural systems for recognizing emotion. Curr. Opin. Neurobiol..

[69] Banissy M.J., Sauter D.A., Ward J., Warren J.E., Walsh V., Scott S.K. (2010). Suppressing sensorimotor activity modulates the discrimination of auditory emotions but not speaker identity. J. Neurosci..

[70] Brecht M., Sakmann B. (2002). Dynamic representation of whisker deflection by synaptic potentials in spiny stellate and pyramidal cells in the barrels and septa of layer 4 rat somatosensory cortex. J. Physiol..

[71] Lausberg H., Sloetjes H. (2009). Coding gestural behavior with the NEUROGES-ELAN system. Behav. Res. Methods.

[72] Rao R.P., Mielke F., Bobrov E., Brecht M. (2014). Vocalization-whisking coordination and multisensory integration of social signals in rat auditory cortex. Elife.

