# Supplementary material for: Tickle contagion in the rat somatosensory cortex
Source: iScience. 2022 Dec 5;25(12):105718. doi: 10.1016/j.isci.2022.105718 (PMC9791364; doi:10.1016/j.isci.2022.105718)
Supplement: Supplementary file 2 — Document S1. Figures S1–S6 and Table S1 [file mmc1.pdf]

iScience, Volume *25*

Supplemental information

Tickle contagion in the rat somatosensory cortex

Lena V. Kaufmann, Michael Brecht, and Shimpei Ishiyama

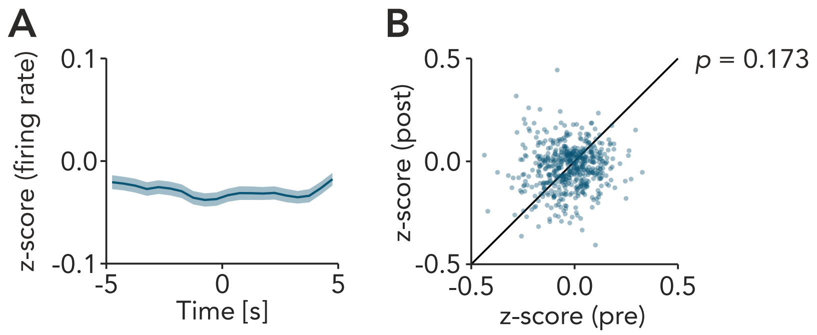

Figure S1. Stability in the baseline firing rate (related to Figure 2).

(A) Peristimulus time histogram of z-scored firing rate (mean ± SEM) at 100 random time points in baseline (545 units, 25 recordings).

(B) Scatter plot shows mean z-score during 'pre' [-5, -1] s vs. 'post' [1, 5] s of (A). *p*-value: signed-rank test.

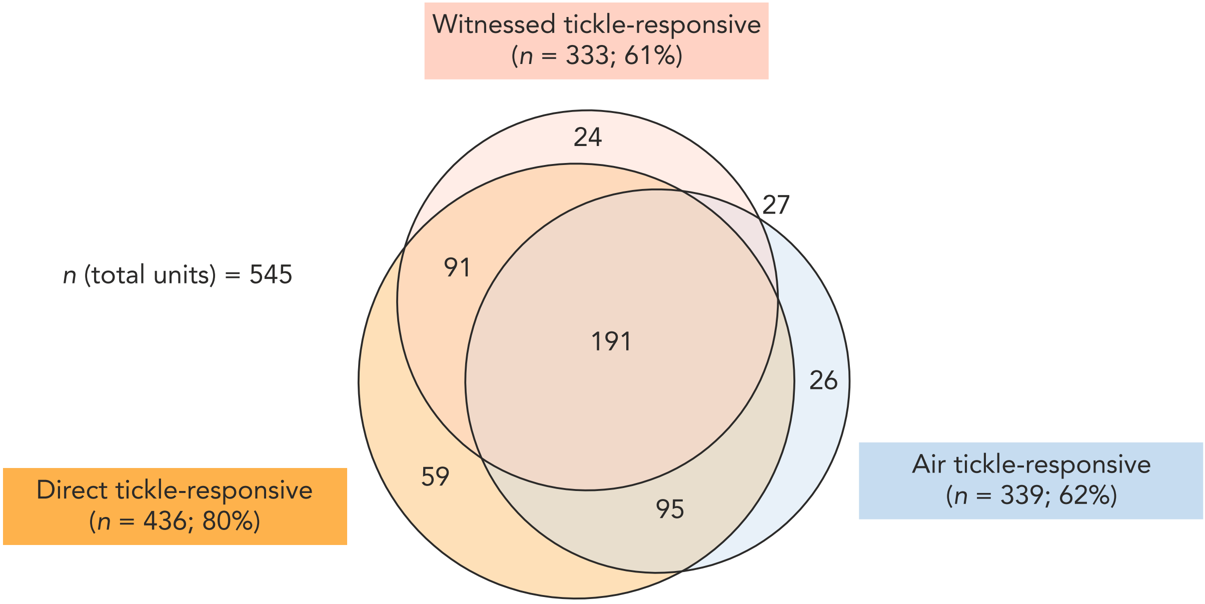

Figure S2. Overview of neuronal response patterns (related to Figure 2).
Venn diagram of response pattern of the total units recorded (*n* = 545 units). Responsiveness was determined as z-scored firing rate during ([1, 5] s) given event is larger than that before ([-5, -1] s) event onset.

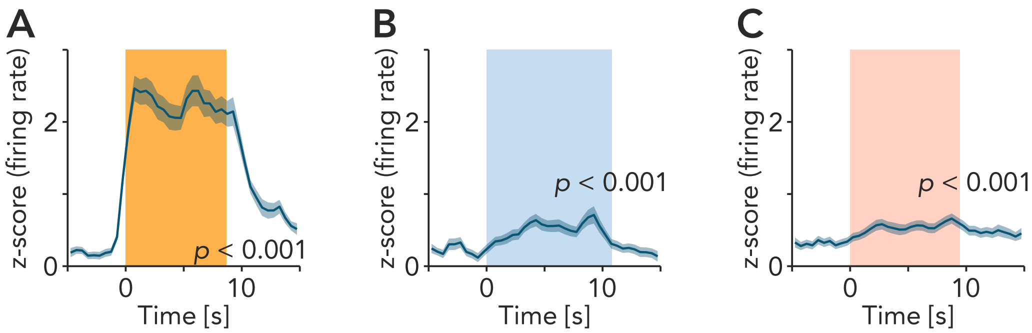

Figure S3. Neuronal response to direct tickling, air tickling and witnessed tickling, with the same axes scaling (related to Figure 2).

(A) Population peristimulus time histogram of z-scored (see methods) firing rate aligned to the onset of direct dorsal tickling (Mean ± SEM; 545 units, 96 events). *p*-value: signed-rank test for z-score before event onset [-5, -1] s vs. after event onset [1, 5] s. Same as Figure 2D.

(B) Same as (D) but for air tickling (545 units, 75 events). Same as Figure 2E but the same axes scaling as (A).

(C) Same as (D) but for witnessed dorsal tickling (545 units, 159 events). Same as Figure 2F but the same axes scaling as (A).

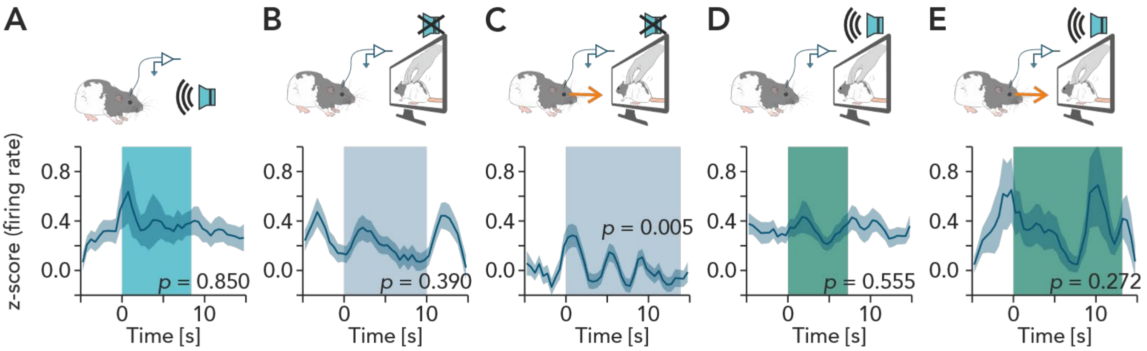

Figure S4. Neuronal response to playback of tickling footage (related to Figure 2&3).

(A) Top, schematic illustration of neuronal recording during audio playback of dorsal tickling footage. Bottom, population peri-stimulus time histogram (PSTH) of z-scored firing rate (mean ± SEM; 0.5 s bin; 479 units; 63 events). Width of the color box indicates mean duration of events. *p*-value: signed-rank test for z-score before event onset vs. after event onset.

(B) Same as (A) but for visual playback of dorsal tickling (320 units, 45 events).

(C) Same as (A) but for watching (turning the head towards) visual playback of dorsal tickling (110 units, 18 events).

(D) Same as (A) but for audio/visual playback of dorsal tickling (111 units, 18 events).

(E) Same as (A) but for watching audio/visual playback of dorsal tickling (148 units, 18 events).

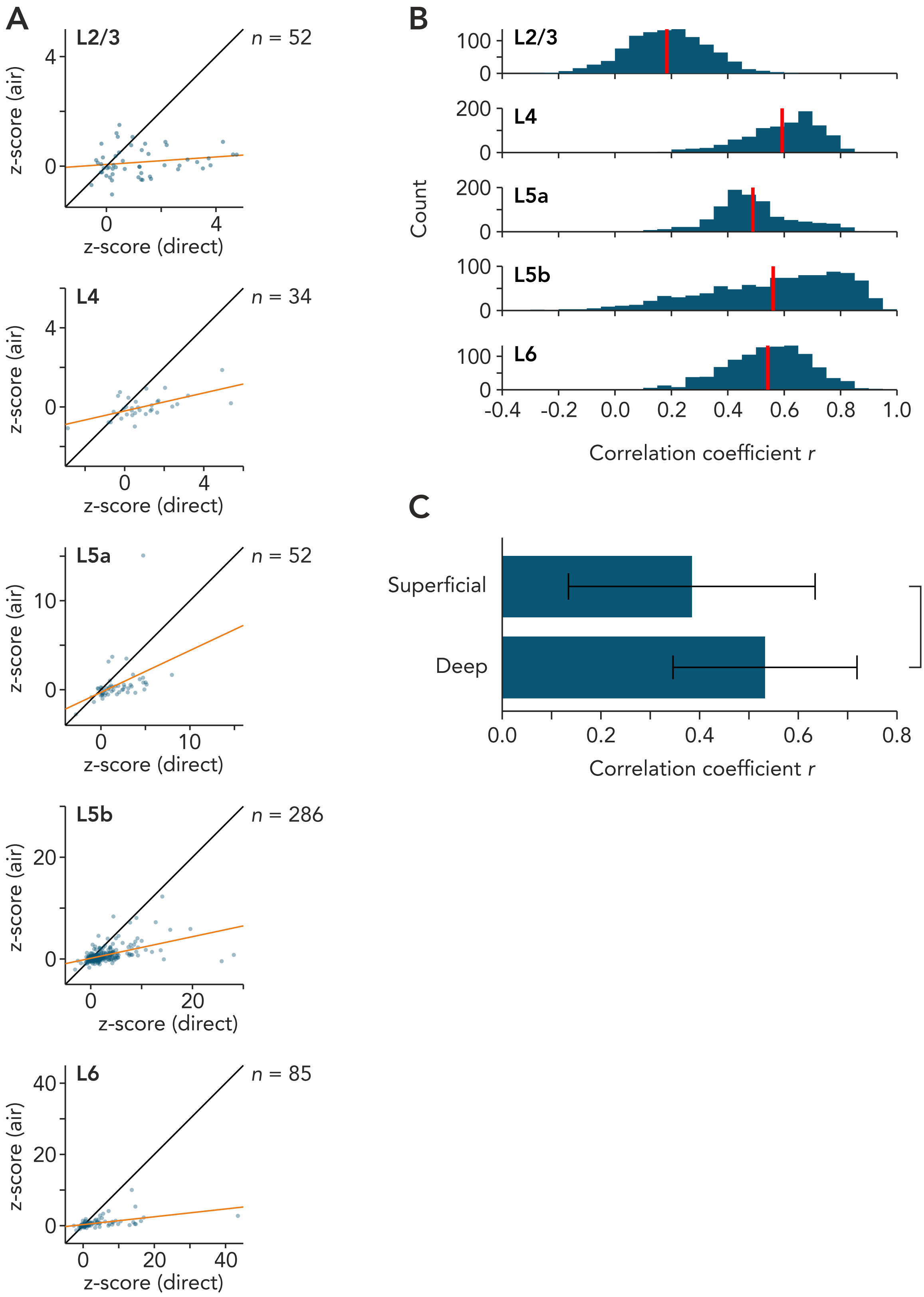

Figure S5. Laminar comparison of correlation between direct and air tickling response (related to Figure 4)

(A) Scatter plots show z-scored firing rate during dorsal tickling (‘direct’) vs. air tickling (‘air’) in layers 2/3, 4, 5a, 5b and 6. Black diagonal lines are unity line. Orange lines are linear fit. *n*: number of units.

(B) Histogram shows distribution of Pearson's correlation coefficient *r* calculated for 1000 times with randomly chosen 20 units, i.e. 60% of layer 4 sample size, in each layer. Red lines: mean. $p < 0.001$ (Kruskal-Wallis test).

(C) Comparison of correlation coefficients in superficial (L2-4) vs. deep (L5-6) layers. Error bars: standard deviation. $p < 0.001$ (rank-sum test).

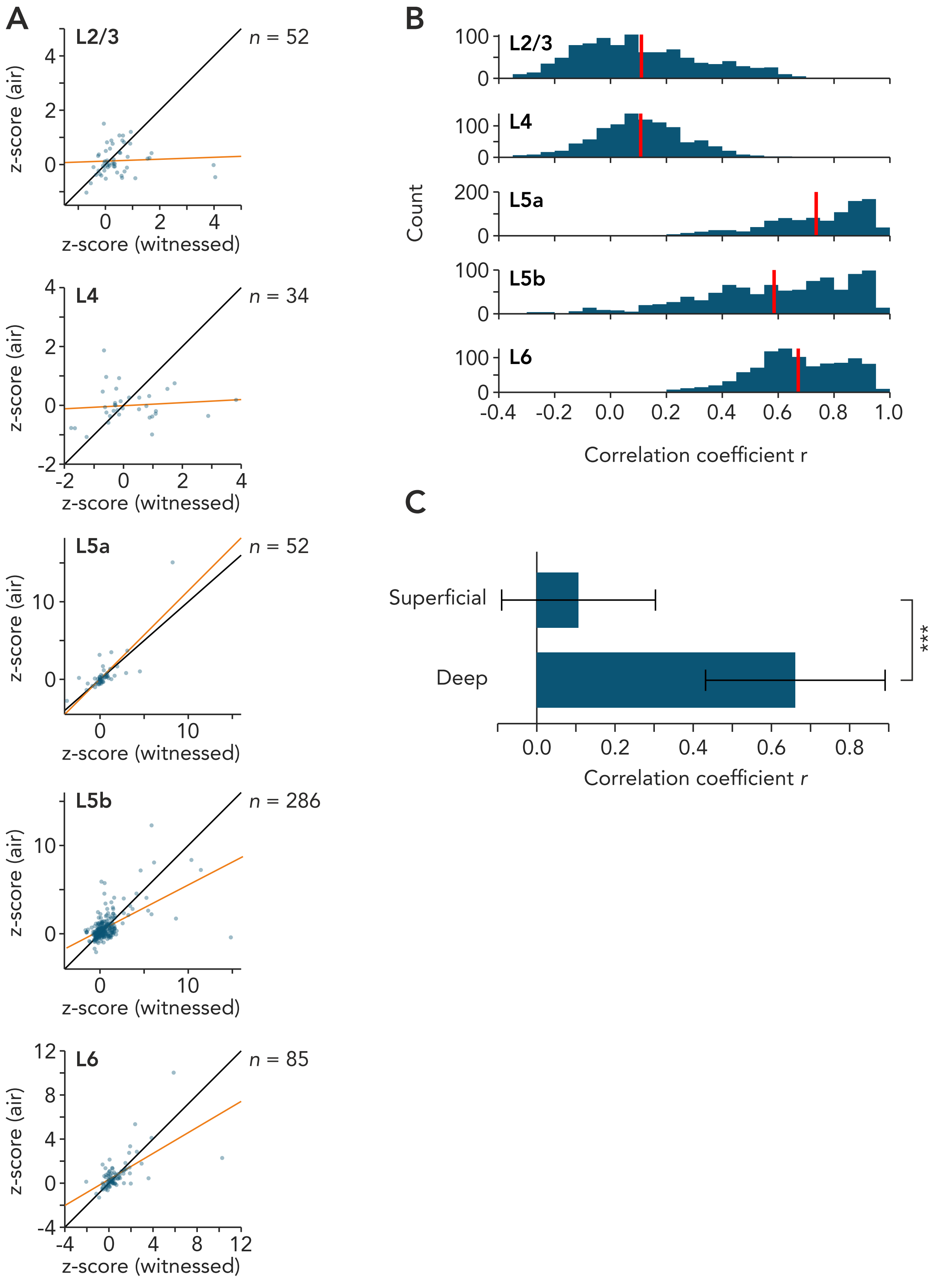

Figure S6. Laminar comparison of correlation between witnessed and air tickling response (related to Figure 4)

(A) Scatter plots show z-scored firing rate witnessed dorsal tickling of demonstrator ('witnessed') vs. air tickling ('air') in layers 2/3, 4, 5a, 5b and 6. Black diagonal lines are unity line. Orange lines are linear fit. *n*: number of units.

(B) Histogram shows distribution of Pearson's correlation coefficient *r* calculated for 1000 times with randomly chosen 20 units, i.e. 60% of layer 4 sample size, in each layer. Red lines: mean. $p < 0.001$ (Kruskal-Wallis test).

(C) Comparison of correlation coefficients in superficial (L2-4) vs. deep (L5-6) layers. Error bars: standard deviation. $p < 0.001$ (rank-sum test).

Table S1. Ethogram of behavioral video analysis (related to Figure 1)

Phase	Category	Description
Baseline	Misc.	First 30 s of the recording.
Break	Misc.	Periods between heterospecific / demonstration events.
Session change	Misc.	Periods when experimental settings were changed (e.g. installing the TV monitor).
Direct dorsal tickling	Heterospecific	Tickling observer's dorsal trunk.
Direct ventral tickling	Heterospecific	Tickling observer's ventral trunk.
Direct flip	Heterospecific	Grabbing the observer's trunk and pinning on the ventral surface, prior to ventral tickling.
Air tickling	Heterospecific	Tickling hand motion in the demonstrator compartment.
A dorsal tickling	Playback	Audio playback of dorsal tickling.
V dorsal tickling	Playback	Visual playback of dorsal tickling.
AV dorsal tickling	Playback	Audio / visual playback of dorsal tickling.
A flip	Playback	Audio playback of flip.
V flip	Playback	Visual playback of flip.
AV flip	Playback	Audio / visual playback of flip.
A ventral tickling	Playback	Audio playback of ventral tickling.
V ventral tickling	Playback	Visual playback of ventral tickling.
AV ventral tickling	Playback	Audio / visual playback of ventral tickling.
A break	Playback	Break in audio playback.
V break	Playback	Break in visual playback.
AV break	Playback	Break in audio / visual playback.
Demo presented	Demonstration	Demonstrator introduced in the demonstrator compartment.
Demo dorsal tickling	Demonstration	Dorsal tickling of demonstrator.
Demo flip	Demonstration	Flipping demonstrator.
Demo ventral tickling	Demonstration	Ventral tickling of demonstrator.
Freudensprung	Spontaneous	Bipedal joy jump of observer.
Demo Freudensprung	Spontaneous	Bipedal joy jump of demonstrator.
Watching demo	Spontaneous	Observer's head was oriented to the demonstrator compartment in the presence of demonstrator without being tickled.
Watching demo tickle	Spontaneous	Observer's head was oriented to the demonstrator compartment during demonstrator tickling.
Watching air tickle	Spontaneous	Observer's head was oriented to the demonstrator compartment during air tickling.
Watching AV playback	Spontaneous	Observer's head was oriented to the demonstrator compartment during audio / visual playback.
Watching V playback	Spontaneous	Observer's head was oriented to the demonstrator compartment during visual playback.
